# Impact of Imperfect Disease Detection on the Identification of Risk Factors in Veterinary Epidemiology

**DOI:** 10.3389/fvets.2019.00066

**Published:** 2019-03-06

**Authors:** Lisa Combelles, Fabien Corbiere, Didier Calavas, Anne Bronner, Viviane Hénaux, Timothée Vergne

**Affiliations:** ^1^UMR ENVT-INRA 1225, Ecole Nationale Vétérinaire de Toulouse, Toulouse, France; ^2^Unité Epidémiologie, ANSES-Laboratoire de Lyon, Université de Lyon, Lyon, France; ^3^Direction générale de l'Alimentation, Bureau de la santé animale, Paris, France

**Keywords:** risk factors, logistic regression, zero-inflated Poisson model, bias, surveillance, sensitivity, bovine abortion

## Abstract

Risk factors are key epidemiological concepts that are used to explain disease distributions. Identifying disease risk factors is generally done by comparing the characteristics of diseased and non-diseased populations. However, imperfect disease detectability generates disease observations that do not necessarily represent accurately the true disease situation. In this study, we conducted an extensive simulation exercise to emphasize the impact of imperfect disease detection on the outcomes of logistic models when case reports are aggregated at a larger scale (e.g., diseased animals aggregated at farm level). We used a probabilistic framework to simulate both the disease distribution in herds and imperfect detectability of the infected animals in these herds. These simulations show that, under logistic models, true herd-level risk factors are generally correctly identified but their associated odds ratio are heavily underestimated as soon as the sensitivity of the detection is less than one. If the detectability of infected animals is not only imperfect but also heterogeneous between herds, the variables associated with the detection heterogeneity are likely to be incorrectly identified as risk factors. This probability of type I error increases with increasing heterogeneity of the detectability, and with decreasing sensitivity. Finally, the simulations highlighted that, when count data is available (e.g., number of infected animals in herds), they should not be reduced to a presence/absence dataset at the herd level (e.g., presence or not of at least one infected animal) but rather modeled directly using zero-inflated count models which are shown to be much less sensitive to imperfect detectability issues. In light of these simulations, we revisited the analysis of the French bovine abortion surveillance data, which has already been shown to be characterized by imperfect and heterogeneous abortion detectability. As expected, we found substantial differences between the quantitative outputs of the logistic model and those of the zero-inflated Poisson model. We conclude by strongly recommending that efforts should be made to account for, or at the very least discuss, imperfect disease detectability when assessing associations between putative risk factors and observed disease distributions, and advocate the use of zero-inflated count models if count data is available.

## Introduction

A disease risk factor is a variable that is associated with an increased likelihood of occurrence of a disease. It is a key epidemiological concept that is used to generate hypotheses about disease origins and to explain disease distributions. Consequently, identifying disease risk factors can help defining effective strategies to monitor, prevent or control epidemics. However, some limits exist since all the possible factors cannot be studied for statistical reasons (1) or practical reasons as the corresponding data is not always available.

Disease risk factors can be identified thanks to observational studies like cohort studies. This approach compares the frequency of disease occurrence between a group of epidemiological units (e.g., animals or herds) exposed to the hypothetical risk factor and a non-exposed group. Alternatively, disease risk factors can be identified thanks to case-control studies, in which the frequency of the hypothesized risk factor is compared between a group of diseased epidemiological units and a non-diseased group. In both of these popular study designs, cases are identified based on a detection process that can be not perfectly sensitive, potentially leading to false negatives. This is particularly true for case-control studies that rely on disease data generated by surveillance systems. Indeed, surveillance systems for endemic diseases, which aim at monitoring disease prevalence or at detecting cases to implement control measures, are generally composed of several surveillance components (like passive surveillance and active surveillance at the abattoir or on farms), all of which being likely imperfectly sensitive (2).

Passive surveillance, which is a continuous surveillance approach based on voluntary reporting of suspect cases by field actors, requires a good detection of diseased animals by farmers and veterinarians followed by a notification to veterinary authorities. An effective passive surveillance component requires a good observation of all the animals of the herd, a knowledge of the warning indicators (increase of mortality, specific clinical signs) and the will to report the suspicion. For some diseases, like the OIE-notifiable diseases (3), reporting suspect cases is mandatory. However, imperfect passive surveillance is very common, and can be explained by different reasons, including economic reasons in the case of stamping-out policies with inappropriate financial compensations (4–6), psychological and social reasons (7–12) and also technical and practical reasons (13). Active surveillance, which is an active search of cases through a pre-defined sampling design, is also imperfect, mainly because it is often based on a sampling of the at-risk population. Moreover, the sensitivity and specificity of diagnostic tests are also imperfect. As a consequence, the sensitivity of the disease observation processes that generate the data that is used to identify disease risk factors is likely to be imperfect and even potentially heterogeneous between epidemiological units, i.e., some cases are more likely to be detected than others.

As a preliminary work of this study, we searched the available literature focused on the identification of animal disease risk factors using surveillance data (see the Supplementary Materials for further details regarding the methodology of this scoping review). It came out that the logistic model was the most common analytical method, used in around 50% of the identified publications. This method, which models the presence or absence of a disease in epidemiological units as a function of some of their characteristics, does not account explicitly for imperfect detection, since epidemiological units with no detected cases are considered as control units, i.e., disease-free units. However, among these papers, more than half discuss the potential bias induced by imperfect detection: for example, the variation of the sensitivity according to the slaughterhouse for detecting a lesion (14) or the influence of human density on the detection of diseased animals (15). In some of the papers that do not used logistic models, authors used zero-inflated (ZI) count models which were claimed to be able to take into account imperfect detection (16, 17). These zero-inflated models, introduced by Lambert, (18), are extensively used in ecology to study the distribution of cryptic animal species (19, 20). ZI count models assume that the number of individuals of a given species observed on a site (in veterinary epidemiology, this could be translated into the number of outbreaks observed in a district or the number of cases observed in a farm) is defined by two successive processes: a binomial process driving the presence or absence of the species on that site and a count process driving the number of observed individuals given the species was present. Therefore, ZI count models in ecology assume two different origins of zeroes: the zeroes related to the absence of the species (“true zeroes”) and, because the observation process is imperfect, those related to the non-detection of any individual of the species despite it is present (“false zeroes”). Consequently, they are relevant when count data is available and when the sensitivity is imperfect, but assume that the specificity is perfect. In epidemiological studies, this is generally a valid assumption since positive tests are often confirmed with another test or combined with clinical suspicions.

The objectives of this paper were (1) to quantify the impacts of an imperfect and potentially heterogeneous case detection sensitivity on the identification of disease risk factors when using logistic models and (2) to emphasize how zero-inflated Poisson (ZIP) models use disease count data to adjust for imperfect detection. These questions were investigated by using simulations and illustrated by revisiting the analysis of the data generated by the French bovine abortion mandatory reporting system, as presented by Bronner et al. (21).

## Materials and Methods

### Simulation Study

In this simulation study, we considered a set of “epidemiological units,” each of them being a cluster of “elementary units” that could be diseased and potentially detected. As an illustration, the epidemiological units could be geographical units (like herds, districts or hexagons) composed of several animals, herds, or villages.

The simulation approach considered two dichotomous factors: a “true” risk factor, referred to as X_1_, which affects the probability of disease presence in epidemiological units, and a second factor, referred to as X_2_, which affects the probability of detecting each diseased elementary unit in diseased epidemiological units (i.e., it affects the detection sensitivity at the elementary unit level). As a consequence, X_2_ is not a risk factor as it is not associated with the probability of the disease to be present in the epidemiological units, but can be considered as a variable that generates an “observational bias.” Fifty percent and 40% of the epidemiological units were associated with the factors X_1_ and X_2_, respectively. These attributions were done independently so that 20% of the epidemiological units had both X_1_ and X_2_, 30% had none and 20% and 30% had only X_2_ and only X_1_, respectively. The disease detection has then been simulated in three successive steps: simulation of the disease presence in epidemiological units, simulation of the true number of cases in diseased epidemiological units and simulation of the observed number of cases in diseased epidemiological units.

#### Simulating Disease Presence

In epidemiological unit *i*, the disease status (D_i_) was considered as a random variable defined by

$$\begin{array}{l} D_{i}\sim Bern\left(prev_{i}\right) \end{array}$$

with

$$\begin{array}{l} prev_{i}=X_{1i}*prev.1+\left(1-X_{1i}\right)*prev.2 \end{array}$$

with prev.1 and prev.2 being parameters defining the probabilities of disease presence in the epidemiological units in which *X*_1_ = 1 and *X*_1_ = 0, respectively. The odds ratio of disease presence [OR(X_1_)] was defined as prev.1^*^(1-prev.2)/[(1-prev.1)^*^prev.2].

Then, the number of cases in diseased epidemiological unit i (C_i_) was considered as a random variable defined by a zero-truncated Poisson distribution (since there cannot be 0 cases in a diseased epidemiological unit) of parameter m, such that

$$\begin{array}{l} \mathrm{Pr}\left(C_{i}=c\right)=\frac{m^{c}}{\left(e^{c}-1\right)*c!} \end{array}$$

with m being the average number of cases in diseased epidemiological units.

#### Simulating Disease Observations

The case detection sensitivity (Se) was assumed to differ between epidemiological units according to the variable X_2_, so that the number of detected cases in epidemiological unit *i* (Y_i_) was considered as a random variable defined by

$$\begin{array}{l} Y_{i}\sim Binom\left(c,Se_{i}\right) \end{array}$$

with c being the true number of cases and

$$\begin{array}{l} Se_{i}=X_{2i}*Se.1+\left(1-X_{2i}\right)*Se.2 \end{array}$$

with Se.1 and Se.2 being parameters defining the case detection sensitivity in the epidemiological units in which *X*_2_ = 1 and *X*_2_ = 0, respectively. Consequently, this simulation framework allowed the presence of false negative epidemiological units, i.e., epidemiological units with at least one case but none detected. Finally, these disease observations were either synthesized as binary data at the level of the epidemiological unit (Y1, presence or not of at least one detected case) to be used in the logistic regression, or considered as a count data (Y2, number of detected cases in epidemiological units) to be used in the zero-inflated Poisson (ZIP) regression. Two hypothetical illustrative examples of this system are presented in the Supplementary Materials.

#### Modeling Disease Observations

The explanatory variables to be used in the logistic and ZIP regressions were the factors X_1_ (associated with the probability of disease presence in epidemiological units) and X_2_ (associated with the likelihood of detecting cases in diseased epidemiological units). The logistic model was defined by

$$\begin{array}{l} logit\left(\mathrm{Pr}\left({Y1}_{i}=1\right)\right)=\alpha_{0}+\alpha_{X1}*x_{1i}+\alpha_{X2}*x_{2i} \end{array}$$

with α_0_, α_X1_, and α_X2_ being the parameters to be estimated and x_1i_ and x_2i_ being the values of X_1_ and X_2_ for epidemiological unit *i*. Note that, for the logistic model, OR(X_1_) = exp(α_*X*1_).

The ZIP model was defined by

$$\begin{array}{l} \mathrm{Pr}(Y2_{i}=y)=\left\{ \begin{array}{l} \left(1-prev_{i}\right)+prev_{i}*e^{-\lambda_{i}}\;if\;y=0\\ prev_{i}*\frac{e^{-\lambda_{i}}*\lambda_{i}^{y}}{y!}\;if\;y>0 \end{array}\right. \end{array}$$

with

$$logit\left(prev_{i}\right)=\beta_{0}+\beta_{X1}*x_{1i}+\beta_{X2}*x_{2i}$$

and

$$\begin{array}{l} \ln\left(\lambda_{i}\right)=\gamma_{0}+\gamma_{X1}*x_{1i}+\gamma_{X2}*x_{2i} \end{array}$$

with β_0_, β_X1_, β_X2_,γ_0_, γ_X1_, and γ_X2_ being the parameters to be estimated and x_1i_ and x_2i_ being the values of X_1_ and X_2_ for epidemiological unit *i*. Note that, for the ZIP model, OR(X_1_) = exp(β_*X*1_). This formulation of the ZIP model makes it clear that it is made of two parts: a “logistic” part which describes the probability of disease presence in the epidemiological units, and a “count” part which describes the number of detected cases in the epidemiological units where the disease is present.

For both the logistic and ZIP regressions, the significant variables were identified based on an automated stepwise backward selection procedure based on the likelihood ratio test (1, 22).

#### Assessing Bias and Accuracy of Model Outputs

For both models, if X_1_ was significantly associated with the probability of disease presence in epidemiological units (*p* < 0.05), the value of the associated odds ratio as estimated by the model OR_model_(X_1_) was recorded and the relative bias (RB) of the odds ratio was calculated as follows:

$$\begin{array}{l} RB=\frac{OR_{model}\left(X_{1}\right)-OR\left(X_{1}\right)}{OR\left(X_{1}\right)} \end{array}$$

with OR(X_1_) being the “true” odds ratio used to simulate the disease occurrence.

#### Simulation Scenarios

The simulations depend on eight parameters that are presented in Table 1. Different scenarios were run to illustrate the effects of different parameters, in situations with a homogeneous sensitivity between the epidemiological units (Scenario 1 and Scenario 2) and in situations with a heterogeneous sensitivity (Scenario 3 and Scenario 4). The number of epidemiological units (N) was set at 10,000 for all scenarios to make sure that not identifying the risk factors is not due to a too small sample size, i.e., to a lack of power of the model. The other parameters (m, prev.2, OR(X_1_), Se.1 et Se.2) were assigned varying values to assess their influence on the model outcomes. For each set of parameter values, 500 simulations were run to compute various summary statistics of model performance, including the probability that the parameters X_1_ and X_2_ are correctly and incorrectly identified as risk factors, respectively, and the relative bias of the odds ratio associated with parameter X_1_. In an exploratory phase, we ran 1,000 simulations but no substantial change in the computed summary statistics could be observed, so 500 simulations were considered a good compromise between computing time and outcome precision. All simulations and analyses were performed using the R software 3.3 version (23).

**Table 1:** Parameter values used in the simulations.

**Parameter**	**Description**	**Scenario 1**	**Scenario 2**	**Scenario 3**	**Scenario 4**
N	Number of epidemiological units	10,000	10,000	10,000	10,000
m	Mean number of cases in diseased epidemiological units	4	1 to 17	4	1 to 17
prev.2	Probability of disease presence in epidemiological units that do not have the factor X_1_	0.2; 0.4; 0.6; 0.8	0.1; 0.2; 0.5	0.1; 0.2; 0.5	0.1; 0.2; 0.5
OR(X_1_)	Odds ratio of the probability of disease presence based on X_1_	1 to 10	2; 5; 10	2; 5; 10	2; 5; 10
Se.1	Sensitivity of detection of diseased elementary units in epidemiological units that have the factor X_2_	0.01, 0.02, …,1	0.01, 0.02, …,1	0.1, 0.2, …,1	0.3; 0.6; 0.9
Se.2	Sensitivity of detection of diseased elementary units in epidemiological units that do not have the factor X_2_	Se.2 = Se.1	Se.2 = Se.1	0.1, 0.2, …,1	0.3; 0.6; 0.9

### Revisiting French Bovine Abortion Surveillance Data

To illustrate with a real example that logistic and ZIP models can lead to significantly different results as suggested by the simulations, we used the French bovine abortion surveillance data and revisited the analysis presented in Bronner et al. (21). Based on a demonstration that only 20 to 30% of abortions are detected visually (24), the authors fitted a zero-inflated model to the number of reported abortions in bovine herds and showed that the probability of reporting at least one abortion in herds where at least one abortion occurred (sensitivity of the reporting at herd-level) was heterogeneous between herds and varied according to the production type and the herd size. Therefore, it provided a useful case study to illustrate the discrepancy between logistic and zero-inflated models when using disease data that have been collected by an imperfect and heterogeneous disease detection process. Note that for the sake of simplicity of this illustrative example, we only used the two explanatory variables that were kept in the final model in Bronner et al. (21) and did not account for other variables that could have been associated with the outcome variable such as the farm biosecurity level for example.

#### Data Sources and Study Population

As described in Bronner et al. (21), all bovine abortions occurring on the French territory have to be reported to the local veterinary authority. All reported abortions are therefore registered in the French national animal health information database (SIGAL). In our study, and similar to Bronner et al. (21), abortion data was extracted from SIGAL and information about the herds (size and type of production) was extracted from the French national cattle register (BDNI). Our study focused on all cattle abortions that occurred in mainland France between August 1st 2010 and July 31st 2011, which was a year without any wave of abortive diseases such as Bluetongue or Schmallenberg disease (21, 25, 26). A random selection of 90% of the farms was used as a model training dataset, while the remaining 10% were used for the evaluation of the fitted models.

#### Statistical Modeling and Model Validation

Logistic and ZIP models were adjusted to the abortion dataset. The epidemiological units were the farms and the elementary units were the animals on the farms. For the logistic model, the response variable was the presence of at least one reported abortion in the farm, while the response variable of the ZIP model was the number of reported abortions per farm. Note that reasons for zero reported abortions in a farm could either be because no abortion occurred or because the farmer did not detect any abortion or because the farmer detected at least one but did not report any. For both models the two putative explanatory variables were the production type (beef, dairy, mixed) and the herd size. Similar to Bronner et al. (21), herd size was categorized into three modalities according to the terciles to allow for non-linear associations with the response variable.

Since we assumed no wave of abortive infectious diseases, the two different models did not account for any spatial dependence of the observations. It is worth stressing that, should there be any evidence or reasons to believe that the condition of interest is contagious and could have spread spatially, including a spatial autocorrelation term in the models becomes necessary (27).

The absence of correlation between the two explanatory variables was verified with a Kendall test (28, 29). The significant variables were identified based on an automated stepwise backward selection process based on the likelihood ratio test (1, 22). The interactions between the two explanatory variables were also tested.

Receiver operating characteristic plots were built using the validation dataset and the areas under the curves (AUC) were calculated to check the ability of the two models to correctly predict the presence of at least one reported abortion in farms.

## Results

### Simulation Study

#### Identification of X_1_ as a Risk Factor

The logistic regression identified correctly and systematically the factor X_1_ as a risk factor, independently of the case detection sensitivity and the mean number of cases in diseased epidemiological units. Similarly, the “logistic” part of the ZIP model correctly and systematically identified X_1_ as a disease risk factor. As a result, the probability of type II error (not identifying a true risk factor) could be considered as very low for both models in our simulations. However, it was noticed that the ZIP model became unstable when the mean number of cases per diseased epidemiological unit was one (results not shown).

#### Identification of X_2_ as a Risk Factor

**Figure 1:**
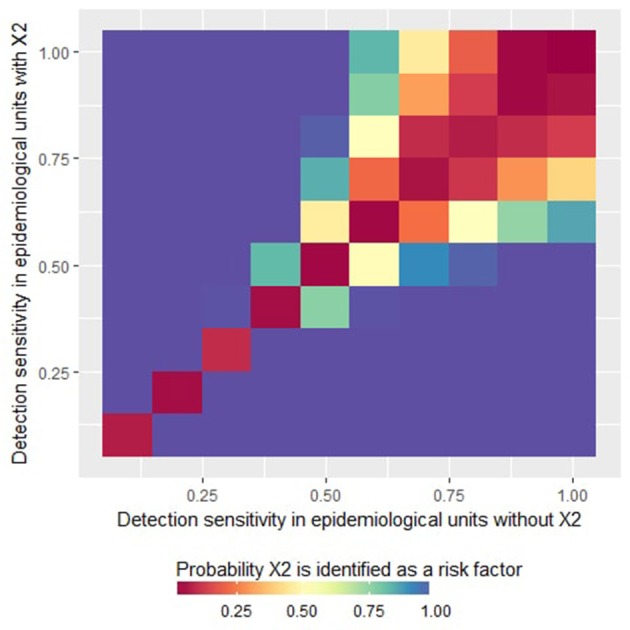
Probability that the factor X_2_ is identified as a variable statistically significantly associated with the presence of at least one reported case (i.e., risk factor for “apparent” presence) by a logistic regression as a function of the detection sensitivity in epidemiological units with *X*_2_ = 0 or *X*_2_ = 1. This figure was done by simulating 500 datasets generated with prev.2 = 0.2, OR(X_1_) = 5 and *m* = 4.

Expectedly, when the detection sensitivity was homogeneous between units (so when X_2_ had no structural influence on the simulations), X_2_ was never identified by the logistic regression as a statistically significant risk factor (see the diagonal on Figure 1). However, when the sensitivity was imperfect and heterogeneous (so when X_2_ influenced the case detection sensitivity), X_2_ was identified as a statistically significant risk factor by the logistic regression, introducing a risk of a type I error (incorrect identification of a variable as a risk factor). More specifically, the risk of a type I error increased with increasing heterogeneity of the detection sensitivity between the epidemiological units. However, for high values of detection sensitivities, the probability of type I error was more limited, even in the presence of heterogeneity (top-right corner of Figure 1). As illustrated in Figure 1, when the detection sensitivity was at least 75% in all the epidemiological units, the probability that the factor X_2_ is wrongly identify as a risk factor was <30%.

**Figure 2:**
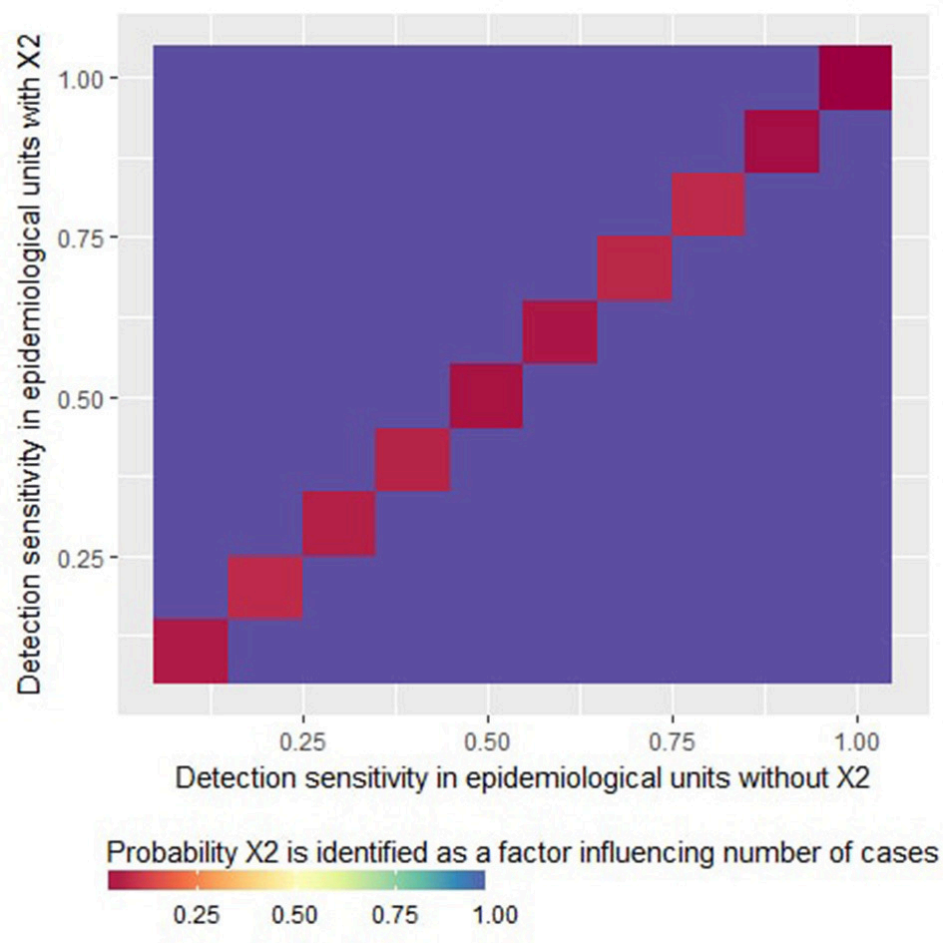
Probability that the factor X_2_ is identified as a variable statistically significantly associated with the average number of reported cases in epidemiological units with at least one case (i.e., risk factor for number of case reports given presence of at least one case) by a zero-inflated Poisson regression as a function of the detection sensitivity in epidemiological units with *X*_2_ = 0 or *X*_2_ = 1. This figure was done by simulating 500 datasets generated with prev.2 = 0.2, OR(X_1_) = 5 and *m* = 4.

The “logistic” part of the ZIP model never identified the factor X_2_ as a risk factor, whatever the case detection sensitivity and the mean true number of cases per diseased epidemiological unit. As a result, the probability of type I error can be considered as very low for the ZIP model in our simulations. Moreover, it can be noted that the “count” part of the ZIP model systematically identified correctly X_2_ as a factor influencing the number of detected cases (see the purple zones on Figure 2). Note that when the sensitivity was homogeneous between units (so when X_2_ had no structural influence on the simulations), X_2_ was never identified as a factor influencing the number of detected cases (see the red diagonal on Figure 2).

#### Bias of the Odds Ratio

**Figure 3:**
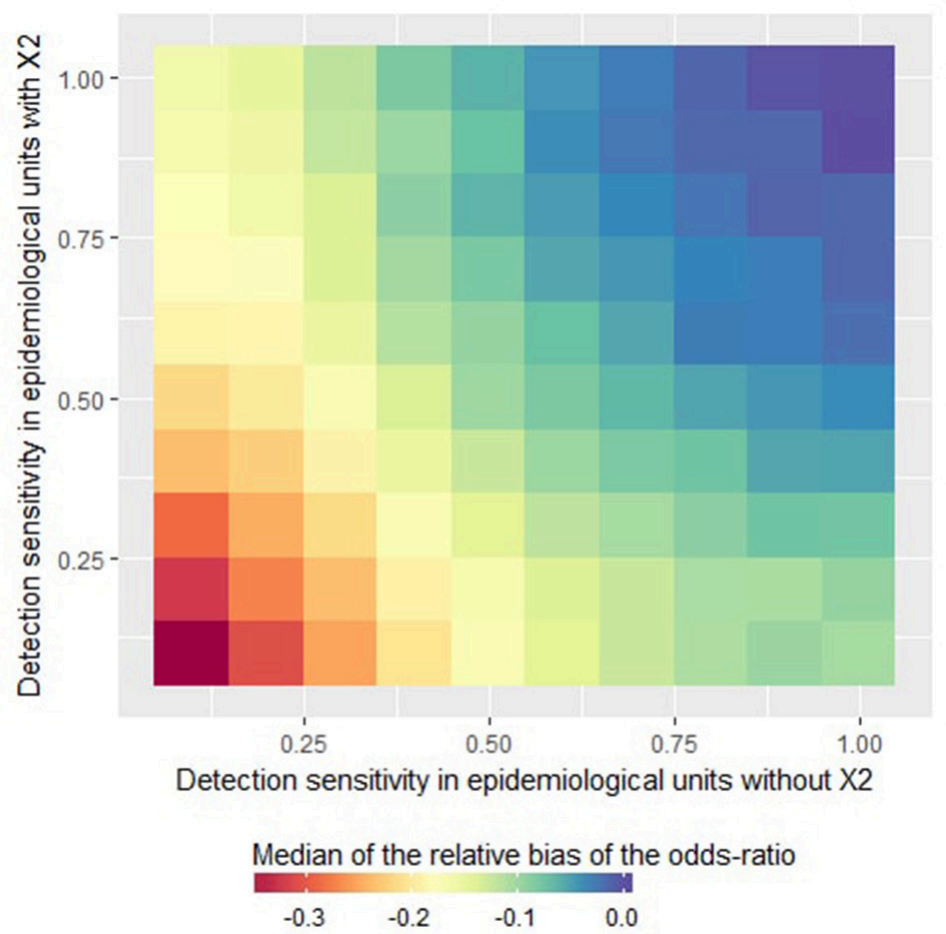
Relative bias of the odds ratio estimated by a logistic regression as a function of the detection sensitivity in epidemiological units with *X*_2_ = 0 or *X*_2_ = 1. This figure was done by simulating 500 datasets generated with prev.2 = 0.2, OR(X_1_) = 5 and *m* = 4.

In the logistic model, imperfect sensitivity led to underestimations of the odds ratio associated with the risk factor X_1_ (Figure 3). For an imperfect but homogeneous sensitivity (i.e., when the sensitivity is less than one but similar in all units), the lower the sensitivity the higher the underestimation (see the diagonal of the Figure 3). In the case of heterogeneous sensitivity, the relative bias decreased when the sensitivity increased in the units where *X*_2_ = 1 even if the sensitivity remained poor in the units where *X*_2_ = 0, and vice-versa. Indeed, as illustrated in Figure 3, when the detection sensitivity was at least 75% in the epidemiological units where *X*_2_ = 1, the odds ratio associated with the risk factor X_1_ was not underestimated by more than 20% even if the detection sensitivity was around 25% in the epidemiological units where *X*_2_ = 0.

The “logistic” part of the ZIP model correctly estimated the odds ratio associated with the risk factor X_1_ (the median of the relative bias was null or almost null), whatever the detection sensitivity and the mean true number of cases per diseased epidemiological unit.

### Bovine Abortion Study

#### Population Characteristics

**Figure 4:**
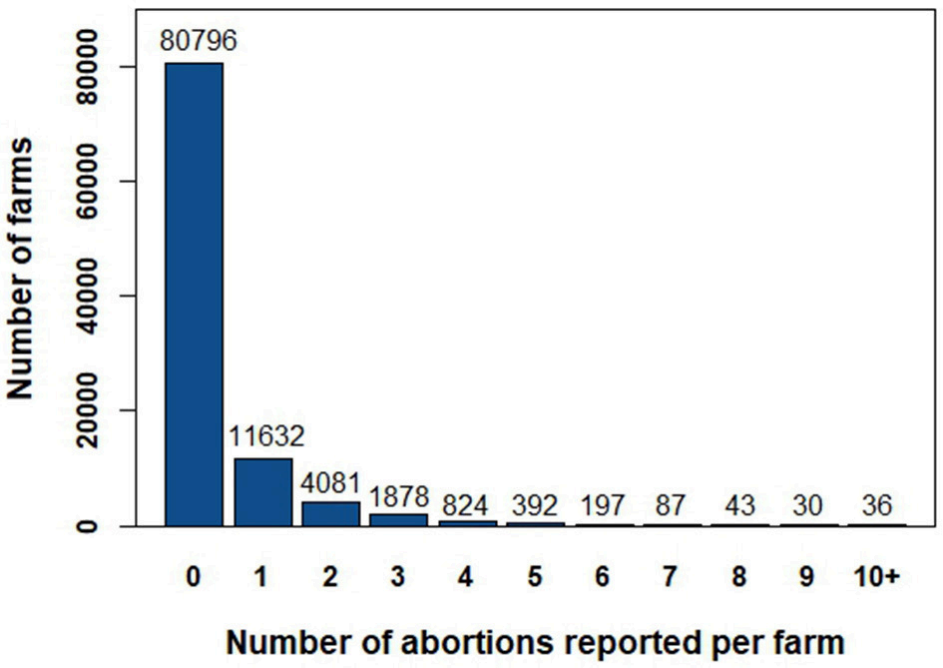
Distribution of the number of reported abortions per farm.

The database included 99,996 farms from 37 departments (French administrative unit). The mean size of the farms was 15,482 bovine-days with a median size of 13,485 bovine-days (minimum = 1; maximum = 276,605). Regarding the production types, 58,979 farms were beef farms (59%), 29,275 were dairy farms (29%), and 11,742 were mixed farms (12%). Overall, 19,200 farms (19%) reported at least one abortion between August 1st 2010 and July 31st 2011. Size distribution, production type distribution and distribution of the number of reported abortions per farm are presented in Table 2 and Figure 4 for the whole database (99,996 farms).

**Table 2:** Distribution of farm characteristics (production type and herd size) according to whether or not at least one abortion was reported during the period of interest.

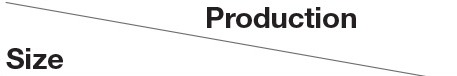	**Farms with no reported abortion**			**Farms with ≥ 1 reported abortion**		
		**Beef**	**Dairy**	**Mixed**	**Beef**	**Dairy**	**Mixed**
≤ 7,686 bovine-day	28,129	2,728	1,028	876	254	43
>7,686 et ≤ 18,586 bovine-day	13,680	9,753	2,236	1,678	4,868	835
>18,586 bovine-day	11,895	6,605	4,742	2,721	5,067	2,858

#### Inferences From the Zero-Inflated Poisson Model

The “count” part of the ZIP model shows that the production type and the size of the French bovine farms were statistically significantly associated with the number of reported abortions in farms where abortions occurred (Table 3). Indeed, compared to beef farms, the number of reported abortions in affected farms was significantly higher in mixed and dairy farms. Similarly, whatever the production type, the bigger the farm the higher the number of reported abortions in affected farms. These factors are therefore likely to influence the probability of reporting at least one abortion in farms with at least one abortion: farmers managing small beef farms with abortions would be less likely to report at least one abortion than farmers managing large dairy farms with abortions, introducing a bias in the abortion presence/absence data at farm level and therefore a potential bias in the outcomes of logistic regressions.

**Table 3:** Results from the bovine abortion zero-inflated Poisson regression.

**Variable**	**Categories**		**OR**	**95%CI**	***p*-value of the LRT**
**“LOGISTIC” PART**					
Production type	Beef		Reference	Reference	
	Mixed		0.90	0.63–1.29	< 0.01
	Dairy		1.97	1.65–2.37	
Herd size	≤7,686		Reference	Reference	
(bovine-day)	>7,686 and ≤ 18,586		2.35	2.00–2.78	< 0.01
	>18,586		3.30	2.81–3.87	
**Interaction**	**Production type**	**Herd size**			
Production type	Mixed	>7,686 and ≤ 18,586	5.78	2.36–14.16	
& Herd size	Dairy	>7,686 and ≤ 18,586	7.86	4.58–13.48	
(bovine-day)	Mixed	>18,586	6.94	2.89–16.69	< 0.01
	Dairy	>18,586	8.79	5.17–14.93	
**Variable**	**Categories**		**IRR**	**95%CI**	***p*-value**
**“COUNT” PART**					
Production type	Beef		reference	reference	
	Mixed		1.60	1.52–1.69	< 0.01
	Dairy		1.75	1.67–1.83	
Herd size	≤7,686		reference	reference	
(bovine-day)	>7,686 and ≤18,586		2.05	1.78–2.35	< 0.01
	>18,586		3.21	2.80–3.69	

*OR, Odds ratio; IRR, incidence rate ratio; 95%CI, 95% confidence interval; LRT, likelihood ratio test*.

The “logistic” part of the ZIP model highlights that the production type and the farm size were factors statistically significantly associated with the probability of having at least one abortion (Table 3). For a given herd size, dairy farms were more likely to have at least one abortion than beef farms. Similarly, for a given production type, medium-size farms and large farms were more likely to have at least one abortion than small-size farms. The interaction between the production type and the herd size highlights that the influence of the herd size varied depending on the production type (Table 3).

#### Inferences From the Logistic Model

The final logistic model included “production type” and “herd size” as statistically significant explanatory variables, as well as the interaction between these two variables (Table 4). For a given herd size, dairy farms were more likely to have reported abortions than beef farms. Similarly, for a given production type, medium-size farms, and large-size farms were more likely to have reported abortions than small-size farms. The statistically significant interaction between the production type and the herd size highlights that the influence of the herd size varies depending on the production type (Table 4).

**Table 4:** Results of the bovine abortion logistic regression.

**Variable**	**Categories**		**OR**	**95%CI**	***p*-value of the LRT**
Production type	Beef		Reference	Reference	
	Mixed		1.35	0.95–1.85	< 0.01
	Dairy		2.96	2.53–3.44	
Herd size	≤7,686		Reference	Reference	
(bovine-day)	>7,686 and ≤18,586		3.91	3.58–4.28	< 0.01
	> 18,586		7.40	6.81–8.04	
**Interaction**	**Production type**	**Herd size**			
Production type	Mixed	>7,686 and ≤ 18,586	11.93	5.56–25.54	
& Herd size	Dairy	>7,686 and ≤ 18,586	16.22	10.77–24.45	< 0.01
(bovine-day)	Mixed	>18,586	19.28	9.13–40.75	
	Dairy	>18,586	24.91	16.66–37.25	

*OR, Odds ratio; 95%CI, 95% confidence interval; LRT, likelihood ratio test*.

### Evaluation of the Models

**Figure 5:**
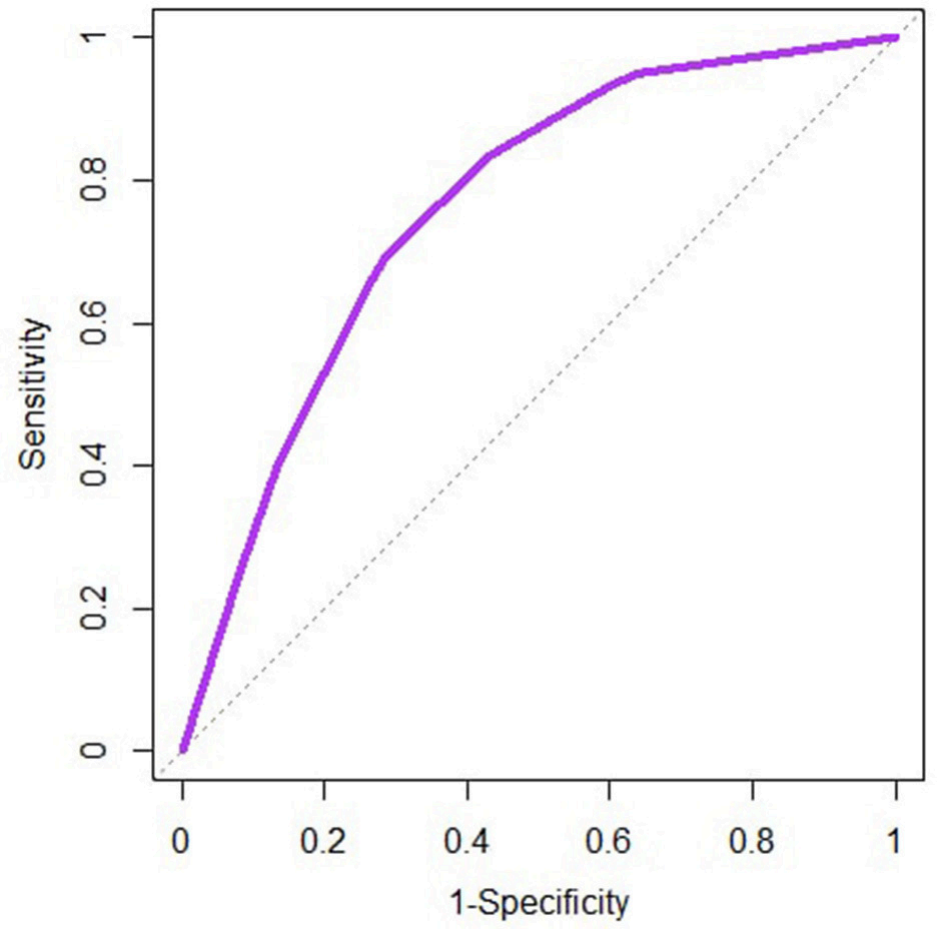
Receiver operating characteristic curve for the logistic model implemented on the bovine abortion dataset. The dotted line represents the diagonal (Sensitivity = 1-Specificity).

The receiver operating characteristic curves are similar between the two models, and only the one for the logistic model is represented on Figure 5. For both models, the area under the curve is 0.76 suggesting an acceptable discriminatory power of each model between the farms with no reported abortion and the ones with at least one reported abortion.

## Discussion

This study illustrates the limitations of logistic regressions for identifying disease risk factors when case detection sensitivity is less than one and potentially heterogeneous between epidemiological units. First, it was shown that imperfect sensitivity has little impact on the risk of a type II error (not identifying a true risk factor) in logistic regressions, but that it could lead to strong underestimations of the odds ratio even with small under-detection issues. The extent of the odds ratio underestimation was shown to increase with decreasing case detection sensitivity and decreasing within-herd prevalence. Finally, this study highlighted that a heterogeneous case detection sensitivity driven by an external variable induces an important risk of a type I error (identifying incorrectly a variable as a risk factor) if this external variable is included as an explanatory variable in logistic regression models and that the risk of this type I error increases with increasing heterogeneity of the detection sensitivity.

Our simulation results suggested that, under the ZIP modeling approach, imperfect and heterogeneous case detection sensitivities had limited impacts on the risk of both type I and type II errors for the identification of risk factors in the “logistic” part of the model. This is due to the “count” part of the ZIP model that enables to explain the average number of detected cases in diseased epidemiological units, allowing for an adjustment of the probability of disease presence in the epidemiological units. This property of the ZIP model made it very popular in ecology to model the distribution of cryptic wildlife populations whose individual detection probability is less than one (19, 20). However, it is worth noting (1) that an increased average number of detected cases in diseased epidemiological units, as modeled by the count part of the ZIP, can be the consequence of either an increased true number of cases in diseased units or of a greater probability of detection of each case (as discussed for the bovine abortion study), and (2) that the count part of the ZIP does not make the difference between these two processes.

Regarding the analysis of bovine abortions in France, the logistic model identified both the production type and the farm size as variables statistically significantly associated with the odds of having at least one reported abortion (Table 3). From this, one cannot say whether this increased odd of having at least one reported abortion is the consequence of an increased chance of having at least one abortion or of an increased chance of reporting at least one abortion in farms with at least one abortion (what could be due to either a higher abundance of abortions in farms with at least one abortion or to a higher probability of detecting and reporting abortions in farms with at least one abortion). The ZIP model identified these two variables both in the “logistic part” and the “count part” of the model (Table 4). Being included in the count part suggests that these variables influence the number of reported abortions [the production type probably because of a closer monitoring of the cows in dairy farms and the farm size probably because of a higher number of opportunities for abortion due to a larger number of cows in large farms, as suggested in Bronner et al. (13)]. Being included in the logistic part of the ZIP (despite the adjustment by the count part) suggests that these variables are also statistically significantly associated with the odds of occurrence of at least one abortion.

Compared to beef herds, dairy herds were found to be associated with a higher probability of occurrence of at least one abortion, but also with a higher average number of reported abortions in herds where at least one abortion occurred (as mentioned just before, this is probably due to the fact that dairy herds are more closely monitored than beef herds so that abortions are more easily detected). This positive association in the count part explains why the “apparent” odds ratio of having at least one abortion in dairy herds as compared to beef herds estimated by the logistic model is greater than the “adjusted” odds ratio of having at least one abortion estimated by the “logistic” part of the ZIP model (Tables 3, 4). The exact same explanation applies to the interpretation of the other differences between the two models. This case study is an eloquent illustration of how quantitative outputs of logistic models can be biased if based on disease data that had been generated by imperfect detection processes. Despite the difference between the quantitative outcomes of the two models, note that the qualitative results are comparable. Indeed, in the two models, both putative risk variables were found to be significantly associated with the presence of at least one abortion in a herd. However, as shown in the simulation study, qualitative results could have been different would the detection heterogeneity had been structured differently.

To identify disease risk factors when disease distribution is observed imperfectly, some authors have suggested using Bayesian hierarchical models that incorporate information on the sensitivity and specificity of disease detection (30). However, this approach requires either an independent study to estimate the sensitivity and specificity parameters or the use of a gold standard applied to a subset of individuals diagnosed with the regular test (30). Capture-recapture methods could also be a useful approach to study disease risk factors when disease observation is imperfect, but they require at least two independent surveillance protocols of the disease of interest (31) or a longitudinal follow-up of the epidemiological units whose disease status is imperfectly observed (32, 33).

## Limitations

The simulation framework did not account for the size of the epidemiological units (e.g., the number of animals in farms), since it was assumed that the number of cases within diseased epidemiological units was small compared to the size of the epidemiological units, what justified the use of a Poisson distribution for the count part of the zero-inflated count model. However, for diseases for which the within-herd prevalence could be greater, it would be necessary to account for the size of the epidemiological units.

Also, the simulation study was designed with the specific aim to provide explicit insights on the limits that the logistic and ZIP models could have in situations with imperfect case detection. As a consequence, it is acknowledged that their design framework sometimes fails to capture the complex reality of field situations. First, the simulation design was based only on two independent factors which were associated either with the probability of disease in the epidemiological units (factor X_1_) or with the probability of case detection in diseased epidemiological units (factor X_2_). Yet, as shown in the analysis of the abortion data, reality is generally more complex with multiple factors being associated with these probabilities and with factors potentially being associated with both probabilities. Then, the simulated population of interest was composed of 10,000 units in order to make sure that the sample size would not jeopardize the power of the model, so that an absence of association could not be attributed to a too small sample size. While national studies based on epidemiological units defined at the farm level (similar to the bovine abortion study) could have sample sizes in the same order of magnitude, studies based on epidemiological units defined at geographical levels such as districts in a country or villages in a region, would likely be associated with much smaller sample sizes. This could lead to a greater risk of type II error than the risk of the imperfect detectability only.

In addition, only logistic regression was used in this study to analyse the aggregated case reports, since it is one of the most popular approaches to analyse binary outcomes. However, it is widely acknowledged that the measure of association of logistic regressions (the odds ratio) can be difficult to communicate, especially to non-epidemiologists and that it can strongly overestimate the prevalence ratio (a measure of association much easier to communicate) when dealing with frequent outcomes (34). To overcome these limitations, alternative models have been advocated to analyse binary outcomes, including Cox, Poisson and log-binomial regressions. An interesting continuation of this work would be to assess the impact of imperfect disease detection on these alternative models.

Finally, the bovine abortion models only included the variables that were significantly associated with the number of abortions per farm in France, as identified by Bronner et al. (21). It is acknowledged that other variables (e.g., production company, biosecurity level, farmer's level of education, etc.) could also contribute to explaining the distribution of abortions and that observations could potentially be spatially dependent, even in the absence of an epidemic of abortive diseases. However, since the objective of these bovine abortion models was to illustrate how logistic and ZIP models could generate different outcomes as demonstrated in the simulation study, the complexity of the two models was purposively kept to a minimum.

## Conclusion

This work showed that, when the detection sensitivity is imperfect, logistic models applied to case reports aggregated at a larger scale (e.g., diseased animals aggregated at farm level or outbreaks aggregated at administrative level) are likely to lead to biased estimates of odds ratios, and even to identify incorrectly as risk factors potential variables influencing the detection sensitivity itself (type I error). Consequently, we strongly recommend that logistic models outputs are systematically discussed with regards to potential imperfect and heterogeneous detectability issues. When count data is available (e.g., number of infected animals in herds), we advocate the importance of not reducing the data to a presence/absence dataset (e.g., presence or not of at least one infected animal in herds) but rather to model it directly using zero-inflated count models.

## Author Contributions

LC contributed to the conception of the study, ran the simulations, conducted the data analysis, interpreted the results, and coordinated the writing of the manuscript. FC contributed to the conception of the study and interpreted the results. DC, AB, and VH provided the bovine abortion data and interpreted the results. TV conceived the study, contributed to the simulations and analysis and interpreted the results. All authors provided substantial contributions to writing or reviewing the manuscript.

### Conflict of Interest Statement

The authors declare that the research was conducted in the absence of any commercial or financial relationships that could be construed as a potential conflict of interest.
