# Supplementary material for: Impact of Imperfect Disease Detection on the Identification of Risk Factors in Veterinary Epidemiology
Source: Front Vet Sci. 2019 Mar 6;6:66. doi: 10.3389/fvets.2019.00066 (PMC6415588; doi:10.3389/fvets.2019.00066)
Supplement: Supplementary file 1 [file Table_1.pdf]

Impact of imperfect disease detectability on the identification of risk factors in veterinary epidemiology

Supplementary Material

Lisa Combelles [1], Fabien Corbiere [1], Didier Calavas [2], Anne Bronner [3], Viviane Hénaux [2], Timothée Vergne [1*]

1 UMR ENVT-INRA 1225, Ecole Nationale Vétérinaire de Toulouse, Toulouse, France
2 Université de Lyon, ANSES - Laboratoire de Lyon, Unité Epidémiologie, Lyon, France
3 Direction générale de l'Alimentation, Bureau de la santé animale, Paris, France

* **Correspondence:** Dr Timothée Vergne, t.vergne@envt.fr

1. Literature review

The outline of the methodological activities undertaken is presented in Figure 1. To identify recent primary research papers aiming at identifying livestock disease risk factors using surveillance data, the search strategy was defined by the following combination of key words: surveillance AND (livestock OR cattle OR cow OR cows OR pig OR pigs OR swine OR sheep OR goat* OR ruminant* OR poultry OR avian) AND (disease* OR health OR infection* OR outbreak*) AND model* AND risk. The search was performed on the 16th of February 2017 on PubMed database. A publication was considered eligible for inclusion if it reported primary research and was published in English after 2005. Note that the objective was not to conduct a systematic review but rather to illustrate the different methods and approaches that are generally used to identify livestock disease risk factors.

Data extracted from the selected publications included: (1) country of the study, (2) disease, (3) studied host species, (4) source of disease data (OIE database, national program,…), (5) epidemiological units (animal, herds, town,…), (6) statistical model used for the identification of risk factors, (7) modelled outcome (disease presence/absence, number of detected cases, prevalence), (8) nature of the covariates, (9) whether the model takes into consideration the quality of the disease detection and (10) whether the authors discuss the quality of the disease detection. A summary of the data extracted from the selected publications is presented in Table 1.

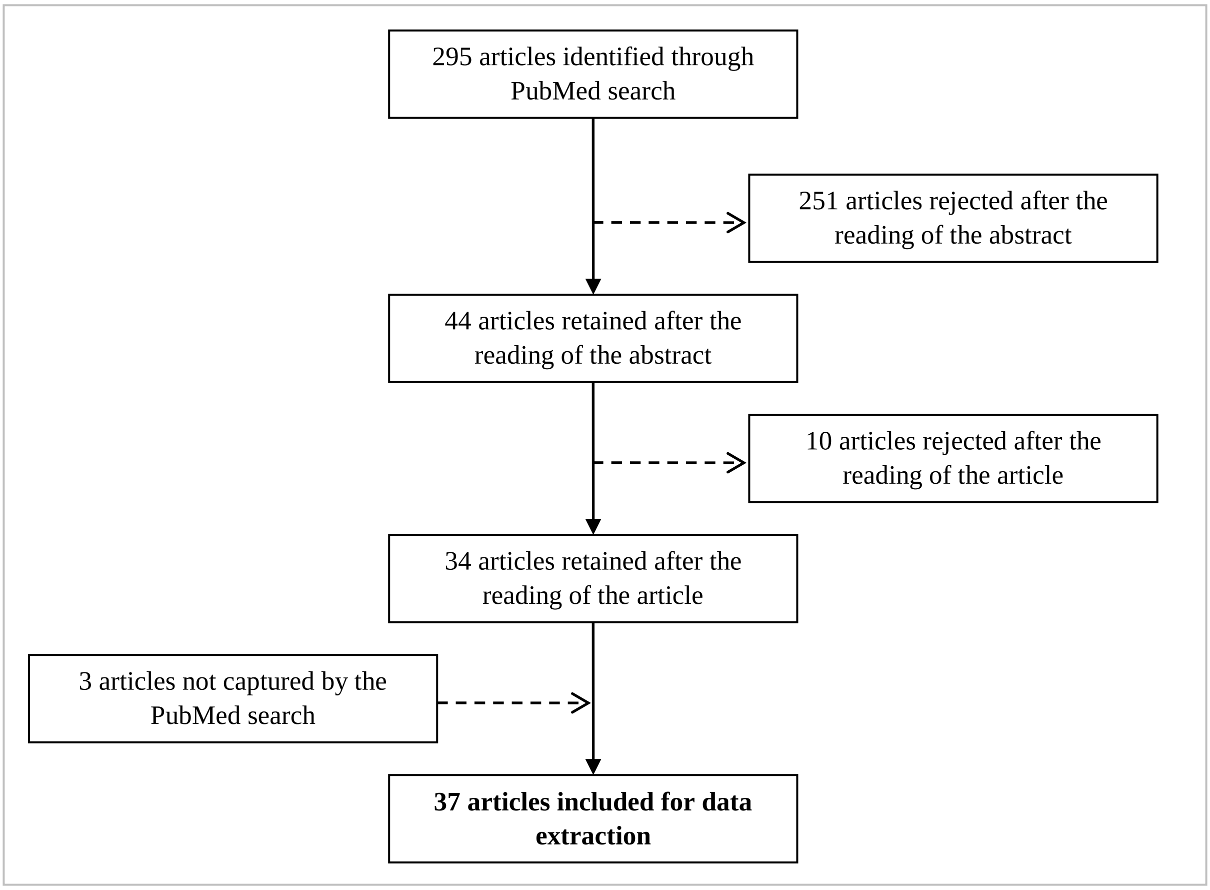

Figure S1: Flow of methodological activities and information through the different phases of the review process.

Table S1: Overview of the studies aiming at identifying livestock disease risk factors using surveillance data (chronological order of publication)

Reference	Disease	Country	Model(s)	Discussion about detection
Abrial et al. (2005)	BSE [1]	France	Poisson	No
Pfeiffer et al. (2007)	HPAI [2] (H5N1)	Vietnam	Logistic	Yes: human factors can help detection (urban *vs* rural)
Porphyre et al. (2008)	Bovine tuberculosis	New Zealand	Poisson	No
Lee et al. (2009)	Bovine brucellosis	South Korea	Logistic	Yes: better detection in large herds
Namata et al. (2009)	Avian salmonellosis	Belgium	Logistic	No
Baptista et al. (2010)	Swine salmonellosis	Portugal	Logistic	Yes: tests sensitivity
Benschop et al. (2010)	Swine salmonellosis	Denmark	Zero-inflated binomial	Yes: sample size
Humblet et al. (2010)	Bovine tuberculosis	Belgium	Logistic	No
Porphyre et al. (2010)	Brucellosis	Armenia	Logistic	Yes: tests sensitivity
Wolfe et al. (2010)	Bovine tuberculosis	Ireland	Logistic	Yes: lesion reports depending of the slaughterhouses
Del Rio Vilas et al. (2011)	Scrapie	Wales	Bayesian hierarchical	Yes: large herds and staff skills
Gulenkin et al. (2011)	African swine fever	Russia	Linear	No
Loth et al. (2011)	HPAI [2] (H5N1)	Indonesia	Logistic; Linear (binomial)	No
Martin et al. (2011)	HPAI [2] (H5N1)	China	Logistic<br>Boosted regression trees	Yes: human factors can help detection (urban *vs* rural)
Rodríguez-Prieto et al. (2012)	Bovine tuberculosis	Spain	Logistic	No
Trevennec et al. (2012)	Influenza H1N1	Vietnam	Zero-inflated binomial	No
Bronner et al. (2013)	Bovine abortion	France	Zero-inflated Poisson<br>Hurdle	Yes: lack of detection and lack of report

Shittu et al. (2013)	Bovine tuberculosis	Great Britain	Logistic	Yes: staff skills and inspection sensitivity (slaughterhouses)
Stevens et al. (2013)	HPAI [2] (H5N1)	Asia	Multicriteria decision analysis	Yes: uncertainty about under report
Dhingra et al. (2014)	HPAI [2] (H5N1)	India	Logistic<br>Boosted regression trees	Yes: lack of detection and lack of report
Korennoy et al. (2014)	African swine fever	Russia	Maximum entropy	No
Martínez-López et al. (2014)	Classical swine fever	Bulgaria	Logistic	No
Pascual-Linaza et al. (2014)	Bluetongue disease	Spain	Logistic	Yes: minimal under report
Sindato et al. (2014)	Rift valley fever	Tanzania	Logistic	Yes: lack of detection and lack of report
Thanapongtharm et al. (2014)	Porcine reproductive and respiratory syndrome	Vietnam	Logistic<br>Boosted regression trees	Yes: highly pathogenic well detected and human factors effect
Vergne et al. (2014)	HPAI [2] (H5N1)	Thailand	Zero-inflated Poisson	Yes: lack of detection and lack of report
Netrabukkana et al. (2015)	Influenza A	Cambodia	Linear	Yes: lack of report
Saksena et al. (2015)	HPAI [2] (H5N1)	Vietnam	Linear<br>Boosted regression trees	Yes: lack of detection and lack of report (urban *vs* rural)
Abdrakhmanov et al. (2016)	Rabies	Kazakhstan	Maximum entropy	Yes: human factors can help detection (urban *vs* rural)
Alkhamis et al. (2016)	HPAI [2] (H5N1)	Middle East	Maximum entropy	Yes: lack of report and human factors effect (urban *vs* rural)
Alkhamis et al. (2016)	Lumpy skin disease	Middle East	Maximum entropy	Yes: lack of report and human factors effect (urban *vs* rural)
Byrne et al. (2016)	Liver fluke infection	Northern Ireland	Logistic<br>Linear	Yes: staff skills and inspection sensitivity (slaughterhouses)
Cowled et al. (2016)	Ovine Johne's disease	Australie	Logistic	Yes: inspection sensitivity
Hayama et al. (2016)	Bovine ephemeral fever	Japan	Logistic	No
Paul et al. (2016)	HPAI [2] (H5N1)	Thailand and Cambodia	Multicriteria decision analysis	Yes: lack of report<br>(in particular in Cambodia)
Vergne et al. (2016b)	African swine fever	Russia	Zero-inflated Poisson	Yes: lack of detection and lack of report
Walsh et al. (2016)	HPAI [2] (H5N1)	Africa, Asia, Europe	Maximum entropy	Yes: lack of report

[1] *BSE: bovine spongiform encephalopathy*

[2] *HPAI: highly pathogenic avian influenza*

2. Hypothetical illustrative examples of the disease presence and observations

a. *Abortions in bovine herds*

In this hypothetical example simplified from Bronner et al. (2013), and using the notations presented in the main text of the manuscript, epidemiological units are bovine herds, Di is a random variable representing whether or not at least one abortion occurred in a herd, Ci is a random variable representing the number of abortions that occurred in a herd where at least one abortion occurred and Yi is a random variable representing the number of abortions that were reported in a herd where at least one abortion occurred. X1 could be the production type (dairy herds could be more likely to experience at least one abortion than beef herds) and X2 could be the herd size (large herds could be associated with a greater average number of reported abortions in abortive herds and therefore with a greater probability of reporting at least one abortion in abortive herds than small herds).

b. *Highly pathogenic avian influenza (HPAI) outbreaks in geographical units*

In this hypothetical example simplified from Vergne et al. (2014), and using the notations presented in the main text of the manuscript, epidemiological units are geographical units (e.g. sub-districts in a country), Di is a random variable representing whether or not at least one highly pathogenic avian influenza outbreak occurred in a sub-district, Ci is a random variable representing the number of outbreaks that occurred in a sub-district where at least one outbreak occurred and Yi is a random variable representing the number of outbreaks that were reported in a sub-district where at least one outbreak occurred. X1 could be the density

of free-grazing ducks (sub-districts with a high density of free-grazing ducks could be more likely to experience at least one HPAI outbreak than sub-districts with a low density) and X2 could be the human population density (sub-districts with a high human population density could be associated with a greater average number of reported outbreaks in affected sub-districts and therefore with a greater probability of reporting at least one outbreak in affected sub-districts than sub-districts with a low human population density).
